# Unilateral Spermatic Cord Metastasis from Gastric Cancer: A Case Report

**DOI:** 10.5812/iranjradiol.8519

**Published:** 2012-11-20

**Authors:** Kang Young Lee, Seong Jin Park, Sung Kyoung Moon, Hyun Cheol Kim

**Affiliations:** 1 Department of Radiology, Soonchunhyang University Hospital, Hoegi-dong, Republic of Korea; 2 Department of Diagnostic Radiology, Kyung Hee University Hospital, Hoegi-dong, Republic of Korea; 3 Department of Radiology, Kyung Hee University Hospital, Sangil-dong, Gangdong, Republic of Korea

**Keywords:** Spermatic Cord, Neoplasm Metastasis, Gastric Cancer

## Abstract

Malignant spermatic cord tumor is rare. Spermatic cord metastasis is less common and the prognosis of these patients is poor. Here we report a case of unilateral spermatic cord metastasis from advanced gastric cancer. A 57-year-old male underwent total gastrectomy due to advanced gastric cancer. Three years later, a painless hard palpable mass in the left inguinal area developed and the pathology revealed a spermatic cord metastasis from stomach cancer.

## 1. Introduction

Malignant lesions of the spermatic cord, both primary and metastatic are extremely uncommon (1) and reports of radiological findings of spermatic cord metastasis are rare. Here we report ultrasonographic, computed tomography (CT) and magnetic resonance imaging (MRI) findings in a case of unilateral spermatic cord metastasis from gastric cancer.

## 2. Case Presentation

**Figure 1:**
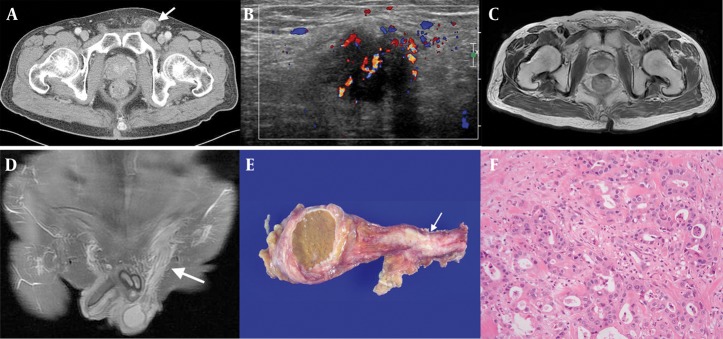
A 57-year-old male with spermatic cord metastasis from advanced gastric cancer. A, Axial contrast-enhanced CT scan showed heterogeneous enhancement and thickening of the left spermatic cord (arrow); B, Scrotal color Doppler ultrasonography revealed an irregular marginated hypoechoic mass with increased vascularity; C, The axial T2-weighted MRI showed a high-signal intensity mass within the left spermatic cord with irregular margins, and the T1-weighted image showed iso-signal intensity of the mass (which is not included in this case report); D, Coronal plane of contrast-enhanced T1-weighted image with fat-suppression showed heterogeneous enhancement (arrow); E, The gross specimen of the spermatic cord revealed an infiltrative whitish solid mass (arrow); F, The light microscope image of the spermatic cord metastasis shows typical glandular structures with malignant cells (hematoxylin and eosin stain × 100).

A 57-year-old male underwent total gastrectomy for an advanced gastric cancer (Borrmann type 2). The histology revealed poorly differentiated adenocarcinoma, penetration of the serosa and regional lymph node metastasis. The patient was followed for 3 years with no evidence of recurrence on computed tomography (CT) scans, endoscopy and PET-CT scans. Three years later, heterogeneous enhancement and thickening of the left spermatic cord was revealed on a follow-up CT scan (Figure 1A). On physical examination, a hard palpable mass was found in the left inguinal area. Ultrasonography revealed an irregular marginated, hypoechoic mass with increased vascularity (Figure 1B), left testicular swelling and mild hydrocele, which were thought to be the result of reactive change to vascular congestion. On MRI scan, the mass appeared as a diffuse thickening of the left spermatic cord with an irregular margin and high-signal intensity on the T2-weighted image, iso-signal intensity on the T1-weighted image and heterogeneous enhancement on the enhanced-T1-weighted image (Figure 1C and D). The patient underwent left radical orchiectomy. The spermatic cord showed an infiltrative solid mass on gross specimen approximately 4 × 1 cm in size (Figure 1E). The pathology revealed atypical glandular structures with malignant cells and was diagnosed as metastatic adenocarcinoma of the spermatic cord from gastric cancer (Figure 1F). The testis and epididymis were histopathologically determined to be free of carcinoma. This patient was followed up with conservative treatment. After 3 months, this patient complained of a palpable mass with pain between the left inguinal area and scrotum. Resection of this mass was performed without a preoperative imaging study. The pathologic result was “metastatic adenocarcinoma probably from the stomach”.

## 3. Discussion

The spermatic cord is an extremely rare site for distant metastasis from a malignant tumor. In adults, most spermatic cord tumors are malignant and have a sarcomatous origin (1, 2). A study of the spermatic cord and epididymis tumors revealed 28.8% were malignant and of those, 8.1% were metastasis (1).

The most common primary origin of a spermatic cord metastasis is the gastrointestinal tract, followed by the pancreas, prostate and the kidneys (3, 4). The colon is the most common primary site originating from the gastrointestinal tract (5); however, in Japan the most frequent primary site is the stomach (3), which may be the result of the high incidence of gastric cancer in that country. Hematogeneous or lymphatic spread are the main routes of metastasis to the spermatic cord; other routes include retrograde extension through the vas deferens, either along its lumen or as a direct extension via the wall of the vas deferens and trans-peritoneal seeding through a patent tunica vaginalis (6). It was not possible to determine if hematogeneous or lymphatic spread occurred in our patient. Clinically, most patients with spermatic cord metastasis present with a painless scrotal mass, although a lower inguinal mass and enlargement of the testis can occur. Hydrocele, hernia and testicular tumors are the most common misdiagnoses of metastatic tumors (7).

Our patient also demonstrated a hard palpable mass on the left inguinal area. In a previous case report, spermatic cord metastasis from lung cancer appeared on the ultrasonogram as a hypoechoic mass in the inguinal canal with an extension into the scrotum and as a solid mass with inner necrosis on CT scan (8). In our case, the radiological findings of spermatic cord metastasis were a mass formation along the spermatic cord and hypo-echogenicity on the ultrasonogram; heterogeneous enhancement on the CT scan and hyper-intensity on theT2-weighted image; iso-intensity on the T1-weighted image and heterogeneous enhancement on the enhanced T1-weighted image. None of the imaging modalities revealed an area of internal necrosis within the mass.

The prognosis for spermatic cord metastasis is poor. In a previous study, the average survival from the time of diagnosis was 9.1 months (4). The use of chemotherapy and radiotherapy for the treatment of tumors with spermatic cord metastasis is controversial (9). The present case report describes ultrasonographic, CT and MRI findings of spermatic cord metastasis seen as a mass or thickening of spermatic cord with an irregular margin.

## References

[1] Beccia DJ, Krane RJ, Olsson CA (1976). Clinical management of non-testicular intrascrotal tumors. J Urol.

[2] Blitzer PH, Dosoretz DE, Proppe KH, Shipley WU (1981). Treatment of malignant tumors of the spermatic cord: a study of 10 cases and a review of the literature. J Urol.

[3] Kanno K, Ohwada S, Nakamura S, Ohya T, Iino Y, Morishita Y (1994). Epididymis metastasis from colon carcinoma: a case report and a review of the Japanese literature. Jpn J Clin Oncol.

[4] Algaba F, Santaularia JM, Villavicencio H (1983). Metastatic tumor of the epididymis and spermatic cord. Eur Urol.

[5] Meacham RB, Mata JA, Espada R, Wheeler TM, Schum CW, Scardino PT (1988). Testicular metastasis as the first manifestation of colon carcinoma. J Urol.

[6] Arlen M, Grabstald H, Whitmore WF (1969). Malignant tumor of the spermatic cord. Cancer.

[7] Kaya C, Tanrikulu H, Yilmaz G, Aker F, Karaman MI (2006). Spermatic cord metastasis as an initial manifestation of non-small cell carcinoma of the lung. Int J Urol.

[8] Dutt N, Bates AW, Baithun SI (2000). Secondary neoplasms of the male genital tract with different patterns of involvement in adults and children. Histopathology.

[9] Mansfield SD, Barakat O, Charnley RM, Jaques BC, O'Suilleabhain CB, Atherton PJ (2005). Management of hilar cholangiocarcinoma in the North of England: pathology, treatment, and outcome. World J Gastroenterol.

